# Catastrophic health expenditure and multimorbidity among older adults in Brazil

**DOI:** 10.11606/s1518-8787.2020054002285

**Published:** 2020-11-27

**Authors:** Gabriella Marques Bernardes, Helton Saulo, Rodrigo Nobre Fernandez, Maria Fernanda Lima-Costa, Fabíola Bof de Andrade

**Affiliations:** I Fundação Oswaldo Cruz Instituto René Rachou Programa de Pós-Graduação em Saúde Coletiva Belo HorizonteMinas Gerais Brasil Fundação Oswaldo Cruz. Instituto René Rachou. Programa de Pós-Graduação em Saúde Coletiva. Belo Horizonte, Minas Gerais, Brasil; II Universidade de Brasília Departamento de Estatística BrasíliaDistrito Federal Brasil Universidade de Brasília. Departamento de Estatística. Brasília, Distrito Federal, Brasil; III Universidade Federal de Pelotas Departamento de Economia PelotasRio Grande do Sul Brasil Universidade Federal de Pelotas. Departamento de Economia. Pelotas, Rio Grande do Sul, Brasil; IV Fundação Oswaldo Cruz Instituto René Rachou Belo HorizonteMinas Gerais Brasil Fundação Oswaldo Cruz. Instituto René Rachou. Belo Horizonte, Minas Gerais, Brasil

**Keywords:** Middle Aged, Aged, Multimorbidity, Socioeconomic Factors, Cost of Illness, Catastrophic Expenditure

## Abstract

**OBJECTIVE:**

To estimate the relation between catastrophic health expenditure (CHE) and multimorbidity in a national representative sample of the Brazilian population aged 50 year or older.

**METHODS:**

This study used data from 8,347 participants of the *Estudo Longitudinal de Saúde dos Idosos Brasileiros* (ELSI – Brazilian Longitudinal Study of Aging) conducted in 2015–2016. The dependent variable was CHE, defined by the ratio between the health expenses of the adult aged 50 years or older and the household income. The variable of interest was multimorbidity (two or more chronic diseases) and the variable used for stratification was the wealth score. The main analyses were based on multivariate logistic regression.

**RESULTS:**

The prevalence of CHE was 17.9% and 7.5%, for expenditures corresponding to 10 and 25% of the household income, respectively. The prevalence of multimorbidity was 63.2%. Multimorbidity showed positive and independent associations with CHE (OR = 1.95, 95%CI 1.67–2.28, and OR = 1.40, 95%CI 1.11–1.76 for expenditures corresponding to 10% and 25%, respectively). Expenditures associated with multimorbidity were higher among those with lower wealth scores.

**CONCLUSIONS:**

The results draw attention to the need for an integrated approach of multimorbidity in health services, in order to avoid CHE, particularly among older adults with worse socioeconomic conditions.

## INTRODUCTION

In recent decades, the concern related to health expenditures has been highlighted globally, considering that such expenditures have grown faster than the Gross Domestic Product (GDP) of most countries, especially in those with low and middle income ^1^ . However, higher national expenditure does not necessarily imply better health conditions or greater equity in access to services ^2^ . In general, health systems should allow people to use the services without incurring financial sacrifice, which is part of the goal of universal health coverage, proposed by the United Nations in their Sustainable Development Goals ^3^ . Although many countries are making efforts in this direction, recent data point to an increasing trend of catastrophic health expenditures ^4^ , those that exceed a percentage of the family's income, consumption or ability to pay ^3^^–^^5^ . There are different cutoff points, varying from 10 to 40%, to define this type of expenditure, depending on the denominator used for this calculation ^3^^–^^5^ .

Data reported by the World Health Organization (WHO) showed that, in 2010, 808 million people (11.7% of the world population) incurred health expenditures that exceeded 10% of the family budget and, for 179 million people (2.6% of the world population), corresponding expenditures exceeded the 25% limit of the family budget ^3^ . In Brazil, between 2007 and 2015, 25.6% of the population incurred health expenditures greater than 10% of the total household expenses or income ^6^ .

Studies indicate greater impact of catastrophic health expenditures in families with lower socioeconomic level ^7^^,^^8^ . In addition, evidence shows positive association between chronic diseases and greater out-of-pocket health expenditures ^9^^,^^10^ . Within the context of population aging, characterized by the predominance and high burden of non-communicable chronic diseases ^11^ , the concern about the financial impact generated by them has been a studied object ^9^^,^^12^ .

The phenomenon of multimorbidity — the simultaneous occurrence of more than one disease in the same individual ^13^ — is also increasingly common, and with a growing trend ^14^ . Older adults with multimorbidity represent a complex demand, since they use health services more frequently ^12^ , raise the costs for health systems ^12^^,^^15^ and increase the chances of catastrophic expenditures for the families ^9^ .

The relation between socioeconomic conditions, chronic diseases and health expenditures in the general population has already been evaluated by different studies ^7^^–^^10^ . However, the association between catastrophic health expenditure in older adults is still little explored, especially in developing countries. In Brazil, this association is unknown, although particularly relevant, considering the fast population aging and the social inequalities in health conditions and in the use of services by this population ^16^ . Thus, the objectives of this study are: (1) to describe the distribution of catastrophic health expenditures (CHE) in a national representative sample of the Brazilian population of older adults; (2) to estimate the association of these expenditures with multimorbidity; and (3) to assess if the socioeconomic conditions interfere in this association.

## METHODS

The data used were from the baseline of the *Estudo Longitudinal de Saúde dos Idosos Brasileiros* (ELSI – Brazilian Longitudinal Study of Aging), conducted in 2015 and 2016 in a sample representative of the non-institutionalized Brazilian population, aged 50 years or older. The ELSI-Brasil used a probabilistic sample selected by stratification processes and conglomerates, in different stages. In the selected households, all residents 50 years or older were eligible for the interview and other procedures. The final sample of the ELSI-Brasil baseline was composed of 9.412 individuals, residing in 70 municipalities in different regions of the country.

In this study, all individuals aged 50 years or older who participated in the baseline survey of the research and who had all the necessary information for the proposed data analysis were considered, totaling 8.347 people. More details on the ELSI-Brasil may be seen on the investigation homepage (elsi.cpqrr.fiocruz.br) and in a previous publication ^17^ .

The ELSI-Brasil was approved by the Research Ethics Committee of the René Rachou Institute of the Oswaldo Cruz Foundation (CAAE:34649814.3.0000.5091) and all participants signed the informed consent form before the interviews.

### Dependent Variable

The dependent variable of the study was catastrophic health expenditure (yes/no), defined as the ratio between out-of-pocket expenditure, relative to all health expenditure incurred by the adults aged 50 years or older in the last 30 days, and the total household income. Individuals whose expenditures reached values equal or greater than 10% or values equal or greater than 25% of the household income were classified as incurring catastrophic expenditures, as previously proposed ^3^^,^^4^ .

The following health expenditures were considered to compose the out-of-pocket expenditure: medical consultation, dentist consultation, hospitalizations, physiotherapist, occupational therapist, speech therapist, psychologist, caregiver/nursing technician, laboratory/image and other examinations, nutritionist and medications. The ELSI-Brasil considered the recall period of 90 days for health expenditure, except for drug expenses, collected based on the last 30 days. Thus, to allow the sum of the values and to calculate the out-of-pocket expenditure in the last 30 days, the expenses evaluated with recall period of 90 days were divided by three. All the variables related to spending and income were deflated for the year of 2015, using the *Índice Nacional de Preços ao Consumidor Amplo* (IPCA – National Broad Consumer Price Index) provided by the Brazilian Institute of Geography and Statistics (IBGE). The purchasing power of 1 real equated, on average, to 95 cents in 2015.

Household income was established by the sum of the incomes of all residents, considering five sources: salary or self-employment; retirement or death pension; *Bolsa Família* (Family Allowance Program), *Benefício de Prestação Continuada* (Continued Installment Benefit Program), alimony or cash donation; rents or leases; and others. The missing data for one or more items represented absence of income in the item in question. Respondents who did not report the exact value of income were asked to inform the closest interval, using the midpoint of the interval as income value. For the last class interval, the value was imputed considering the median gain of the other residents with income in this range, who informed their income in the open question.

### Covariates

Multimorbidity, defined as the presence of two or more chronic health conditions ^13^ , was the main independent variable. Chronic diseases were selected from a list with 17 diseases, including: high blood pressure, diabetes, high cholesterol, heart attack, angina, heart failure, stroke, asthma, chronic obstructive pulmonary disease, arthritis or rheumatism, osteoporosis, spine problem, depression, cancer, chronic kidney failure, Parkinson's disease and Alzheimer's disease. The presence of these diseases was evaluated through self-report, using the question “Has any doctor ever said you have...”.

Other dependent variables included: age (50–59, 60–69, 70–79 and 80 years or older); sex (female and male); proportion of older adults in the household; education (no study, 1–3, 4–7 and 8 or more years of study); marital status (with or without marital relationship); wealth score (1st, 2nd, 3rd, 4th and 5th quintiles); average number of drugs used; private health insurance (yes or no); and functional limitations (yes and no).

The wealth score, categorized in quintiles, was defined by analyzing main components ^18^ , using information on family ownership of durable goods and housing characteristics: household goods (internet, television, cable television, refrigerator, washing machine, dishwasher, dryer, computer, desk phone, cell phone, microwave, motorcycle, car) and domestic characteristics (maid, masonry or wooden wall, piped water, paved street, bathroom).

Functional limitation was evaluated according to the report of difficulties to perform one of more basic activities of daily living (crossing from one room to another, getting dressed, bathing, eating, lying down and/or getting out of bed and using the toilet).

### Data Analysis

A descriptive analysis of the study variables and of the proportion of health expenditures in relation to out-of-pocket was performed. Univariate logistic regression was used, followed by multiple logistic regression, to assess the association between catastrophic expenditure and each independent variable. The multiple model included all the variables with p-value less than 0.20, and those significantly associated with the outcome remained in the final model. Results were expressed by odds ratio and respective confidence intervals (95%CI). The adjusted probabilities of catastrophic expenditure among individuals with multimorbidity according to the wealth score were calculated from including the interaction term between multimorbidity and wealth score. Data analysis was performed with the Stata 14.0 software (StataCorp College Station, United States), considering sample parameters and weights of the individuals.

## RESULTS

Table 1 shows the descriptive analysis of the sample, composed mostly of women (54%), adults in the age group between 50 and 59 years (46.9%), with marital relationship (63.2%) and 8 years or more of study (37.4%). Most of the studied population had multimorbidity (63.2%), no functional limitation (84.4%) and no private health insurance (74.1%). The prevalence of CHE was 17.9% for the 10% cutoff point of and 7.5% for the 25% cutoff point of household income.

**Table 1:** Sociodemographic and health characteristics of the research participants (Brazilian Longitudinal Study of Aging), 2015–2016.

Variables		%	95%CI
Sex			
	Female	54.0	51.1–56.9
	Male	46.0	43.1–48.9
Age (years)			
	50–59	46.9	42.6–51.3
	60–69	30.0	28.1–31.9
	70–79	15.8	14.0–17.8
	80+	7.3	6.0–8.8
Marital status			
	No marital relationship	36.8	33.9–39.8
	With marital relationship	63.2	60.2–66.1
Education			
	No study	12.3	10.0–14.9
	1–3 years	18.7	17.1–20.4
	4–7 years	31.6	29.1–34.2
	8+ years	37.4	34.6–40.4
	Proportion of older adults in the household	70.7	69.2–72.2
Multimorbidity			
	No	36.8	34.6–39.1
	Yes	63.2	60.9–65.4
	Medications (mean)	2.2	2.1–2.3
Functional limitation			
	No	84.4	83.0–85.7
	Yes	15.6	14.3–17.0
Private Health Insurance			
	No	74.1	1.4–76.7
	Yes	25.9	23.3–28.6
Catastrophic health expenditure			
	10% cutoff point	17.9	16.4–19.5
	25% cutoff point	7.5	6.6–8.4

The evaluation of components of health expenditure pointed that spending with medications represented the greatest proportion of out-of-pocket expenditure (65.1%), followed by spending on dentists and with medical consultations ( Table 2 ).

**Table 2:** Mean proportion of health expenditures in relation to out-of-pocket of older adults in Brazil (Brazilian Longitudinal Study of Aging), 2015–2016.

Expenditure	Out-of-pocket	
	%	95%CI
Medication	65.1	63.0–67.3
Dentist	12.2	10.7–13.7
Medical consultation	11.1	9.8–12.4
Examination	8.7	7.5–10.0
Physiotherapist	1.4	1.0–1.7
Caregiver	0.8	0.4–1.2
Hospitalization	0.5	0.3–0.7
Psychologist	0.5	0.3–0.6
Nutritionist	0.2	0.1–0.3
Occupational therapist	0.0	0.0–0.07
Speech therapist	0.0	0.0–0.1

In the univariate analysis, all variables were significantly associated with catastrophic expenditure in both cutoff points, except for education for both points and physical activity for the 25% cutoff point of household income ( Table 3 ).

**Table 3:** Univariate analysis between catastrophic expenditure and independent variables (Brazilian Longitudinal Study of Aging), 2015–2016.

Variables		Catastrophic expenditure	
		≥ 10% OR (95%CI)	≥ 25% OR (95%CI)
Sex (ref. a : female)			
	Male	0.59 (0.51-0.70) b	0.53 (0.42–0.68) b
Age (years)		1.03 (1.02–1.04) b	1.02 (1.01–1.04) b
Marital status (ref. no)			
	Yes	0.73 (0.64-0.82) b	0.59 (0.51-0.70) b
Years of study		0.99 (0.98–1.01)	1.00 (0.98–1.02)
Wealth score (ref. a : 1st quintile)			
	2nd quintile	1.28 (1.02–1.60) c	1.36 (1.03–1.79) c
	3rd quintile	1.16 (0.93–1.43)	1.09 (0.81–1.46)
	4th quintile	0.94 (0.72–1.23)	0.89 (0.63–1.24)
	5th quintile (richest)	0.92 (0.69–1.23)	1.04 (0.72–1.49)
Proportion of older adults in the household		1.01 (1.01–1.01) b	1.01 (1.00–1.01) b
Physical activity (ref. a : no)			
	Yes	0.79 (0.67-0.93) d	0.84 (0.68–1.04)
	Multimorbidity (ref. a : no)		
	Yes	2.45 (2.11–2.84) b	1.83 (1.47–2.28) b
	No. of medications	1.34 (1.30–1.39) b	1.27 (1.22–1.32) b
Functional limitation (ref. a : no)			
	Yes	2.45 (2.15–2.78) b	2.26 (1.78–2.87) b
Private health insurance (ref. a : no)			
	Yes	1.36 (1.15–1.60) b	1.49 (1.17–1.88) b

ref.ᵃ: reference category

b p < 0.001

c p < 0.05

d p < 0.01

Table 4 shows the results of the factors associated with CHE. After adjusting the model, older females, with lower education, functional limitation and who had private health insurance were more likely to incur CHE, for both cutoff points. Only the association between 4-7 years of education and expenditure equal or greater than 25% of household income and the association between wealth score for the same cutoff point were not statistically significant after adjusting the model. The presence of multimorbidity increased in 95% the chance of CHE equal or greater than 10%, in addition to increasing by 40% the chance of CHE equal or greater than 25% of household income. The results also pointed out that older adults belonging to the highest wealth score are 5% less likely to incur catastrophic expenditure for the 10% cutoff point of household income ( Table 4 ).

**Table 4:** Multiple logistic regression model for factors associated with catastrophic health expenditure (Brazilian Longitudinal Study of Aging), 2015-2016.

Variables		Catastrophic expenditure	
		10% OR (95%CI)	25% OR (95%CI)
Sex			
	Male	0.69 (0.58–0.81) a	0.59 (0.45–0.76) a
Age		1.02 (1.01–1.03) a	1.01 (1.01–1.01) b
Education (8+ years)			
	Did not study	0.65 (0.50–0.85) b	0.64 (0.45–0.91) c
	1–3 years	0.71 (0.57–0.89) b	0.75 (0.59–0.96) c
	4–7 years	0.79 (0.67–0.93) d	0.82 (0.65–1.04)
Wealth score		0.95 (0.92–0.98) b	0.96 (0.90–1.01)
Proportion of older adults in the household		1.01 (1.00–1.01) a	1.01 (1.00–1.01) b
Multimorbidity			
	Yes	1.95 (1.67–2.28) a	1.40 (1.11–1.76) b
Functional limitation			
	Yes	2.06 (1.79–2.37) a	2.04 (1.62–2.56) a
Private health insurance			
	Yes	1.28 (1.07–1.53) b	1.40 (1.11–1.76) b

a p < 0.001

b p < 0.01

c p < 0.05

Figure 1 shows the adjusted probabilities of CHE according to the presence of multimorbidity and wealth score. In the two cutoff points, there were significant differences between the probability of catastrophic expenditures according to the presence of multimorbidity and wealth score. CHE probabilities were higher among individuals with multimorbidity and lower wealth scores, for both cutoff points.

**Figure:**
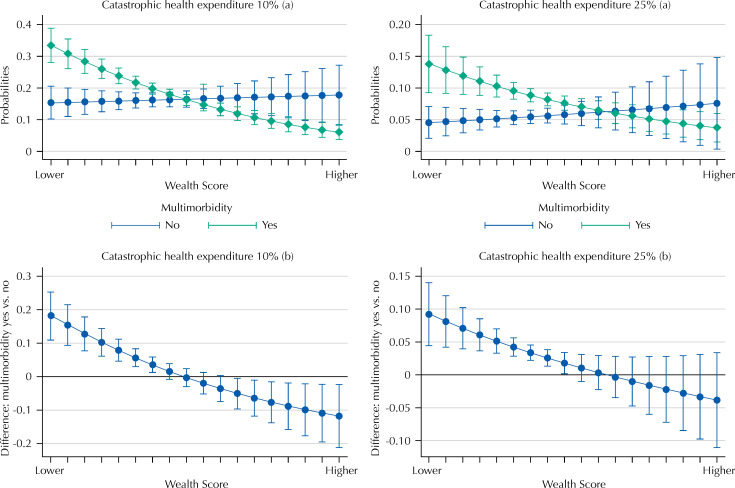
Adjusted probability of catastrophic health expenditure among older adults with and without multimorbidity according to the wealth score (Brazilian Longitudinal Study of Aging), 2015–2016. (a) Adjusted probabilities of catastrophic expenditure among older adults with and without multimorbidity according to wealth score for each cutoff point. (b) Differences between the probabilities of catastrophic expenditure among older adults with and without multimorbidity according to wealth score for each cut-off point.

## DISCUSSION

This study verified a significant impact of health expenses on the household income of older adults. Approximately one fifth of the individuals analyzed incurred CHE exceeding 10% of the household income, and 8% of the individuals incurred CHE when the 25% cutoff point was used. Spending with medication was the main health expenditure. CHE was significantly associated with multimorbidity, which also potentialized the effects of socioeconomic inequalities, regardless of the cutoff point used.

The estimated prevalence of CHE for both cutoff points is higher than that found in the total population of wealthy countries and Latin American and Caribbean countries ^4^ , where the estimated CHE reached 13.4% in 2000, considering the total expenditure on family health for the cutoff point of 10% of family consumption ^4^ . Among wealthy countries, a study performed with Australians aged 55 years or older verified that 11.8% and 5.1% incurred CHE for the 10% and 25% cutoff points, respectively ^9^ . Given the WHO estimate of 25.6%, between 2007 and 2015, which considered the Brazilian population as a whole ^6^ , the prevalence of CHE in older adults in the country for the 10% cutoff point may be considered high.

The higher prevalence of CHE among older adults, in relation to the total population, has been described in literature ^19^ , and may be explained by the differences in types and frequency of spending among young families, when compared to older adult families. In general, most health expenditure relates to spending with medications and medical consultations ^20^ . Among older adults, in addition to those expenses being higher, their proportion in relation to income is also greater ^15^ .

Corroborating a previous study ^9^ , it was found that individuals with multimorbidity were more likely to have catastrophic expenses, regardless of the cutoff point. McRae et al. ^9^ not only observed positive association between multimorbidity and CHE among older adults, but also verified a gradient in this relation: each additional chronic disease increased in 46% the chance of health spending exceeding 20% of the income ^9^ . Among the reasons for this association, we can mention the greater use of services by individuals with multimorbidity ^12^ , which is related to the complexity of care, such as the need to use several medications ^21^ . Individuals with multimorbidity may spend, on average, 10 times more on health than those without it ^10^ .

Our results also showed that socioeconomic inequality is related to CHE, a relation consistently described in literature ^8^^,^^22^ . In Brazil, the occurrence of CHE for total population was 5.2 times higher among the poorest, according to the National Economic Index, and 4.2 times higher among individuals with less schooling, considering complete years of study ^8^ . This study advances by showing that the inequalities associated with CHE in older adults show distinct patterns, when evaluated as a function of multimorbidity, which potentializes the effects of these inequalities. Regardless of the cutoff point used, the probability of CHE was greater among individuals with multimorbidity and belonging to the lowest wealth scores, when compared to individuals without multimorbidity, and this difference was significantly attenuated with increased socioeconomic status.

Both results reinforce the importance of investing in achieving universal health coverage that ensures the access to quality care and services, without catastrophically compromising the family budget ^3^ . In Brazil, the maintenance of the Unified Health System (SUS) is essential for this goal to be achieved, since, throughout its 30 years of existence, the system was effective in reducing inequalities in the health access and conditions of the population ^23^ , especially among individuals with chronic diseases, considering that more than 70% of Brazilian older adults depend exclusively on SUS ^24^ . In addition, data from the most recent national survey on access to medications in Brazil showed that 67.7% of Brazilian adults and older adults with chronic diseases who had total access to treatment, obtained some medications, and 47.5% obtained all their medications, free of charge ^25^ .

Although access to free medicines in the SUS is greater among low-income individuals, middle-income people also often access public pharmaceutical assistance ^26^ . This may be related to the type of private health insurance acquired by this portion of the population. These insurances commonly have limited coverage for medications, home care, physiotherapy and other services, causing individuals who have this type of private health insurance to resort to the public system to ensure access to these services ^27^ . A recent study demonstrated the exclusive use of SUS to obtain medications by 29.3% of the individuals with chronic diseases who had a private health insurance, and among 34.4% of those with these diseases who belonged to the highest economic classification ^27^ . This may be one of the explanations for the fact that private health insurance does not constitute a factor of protection against catastrophic health expenditures ^7^ , and may even increase the occurrence of this outcome, as the results of this study indicated. As in Brazil, other recent studies conducted in countries such as Mexico ^28^ and China ^29^ have shown that public health programs decrease the risk of catastrophic expenditure.

Among the strengths of this study, we highlight the use of a measure of health expenditure composed by a comprehensive number of expenses, usually unavailable in population studies, enabling a more specific analysis of spending by older adults in Brazil. However, the self-report of chronic diseases may interfere with the estimation of multimorbidity, although this measure is considered valid in epidemiological studies, without the occurrence of socioeconomic bias ^30^ . The fact that this study used out-of-pocket expenditure in the numerator, as well as household income in the denominator, to the detriment of total health expenditure and the family's ability to pay, may have reduced the percentage of CHE. Although such measures are widely used in research, literature shows no consensus on the best form of calculation. The cross-sectional design is another limitation of this study, as it prevents the analysis of temporal relationships between the variables. The recall period of the measures used to calculate the CHE, restricted to 30 to 90 days, may also have interfered with the magnitude of the outcome.

The results of this study help to understand catastrophic health expenditure among older adults in Brazil, which can be potentialized by the presence of multimorbidity and among individuals with low socioeconomic status. Considering population aging, along with an increased prevalence of multimorbidity ^14^ , health systems need to adapt to this demand, ensuring the access and quality of their services, especially to the less advantaged population groups. Future research is necessary to monitor our findings and to investigate the influence of specific combinations of diseases on CHE occurrence, in order to contribute to the evaluation of the impact of universal health coverage policies on reducing inequalities.
