# Supplementary material for: Catastrophic health expenditure and multimorbidity among older adults in Brazil
Source: Rev Saude Publica. 2020 Nov 27;54:125. doi: 10.11606/s1518-8787.2020054002285 (PMC7703545; doi:10.11606/s1518-8787.2020054002285)
Supplement: Translation

# Translation: Gastos catastróficos em saúde e multimorbidade entre adultos mais velhos no Brasil

**Authors:** Gabriella Marques Bernardes, Helton Saulo, Rodrigo Nobre Fernandez, Maria Fernanda Lima-Costa, Fabíola Bof de Andrade

**Affiliations:** I Belo HorizonteMinas Gerais Brasil Fundação Oswaldo Cruz. Instituto René Rachou. Programa de Pós-Graduação em Saúde Coletiva. Belo Horizonte, Minas Gerais, Brasil; II BrasíliaDistrito Federal Brasil Universidade de Brasília. Departamento de Estatística. Brasília, Distrito Federal, Brasil; III PelotasRio Grande do Sul Brasil Universidade Federal de Pelotas. Departamento de Economia. Pelotas, Rio Grande do Sul, Brasil; IV Belo HorizonteMinas Gerais Brasil Fundação Oswaldo Cruz. Instituto René Rachou. Belo Horizonte, Minas Gerais, Brasil

**Keywords:** Idoso, Multimorbidade, Fatores Socioeconômicos, Gasto Catastrófico

## Abstract

**OBJETIVO:**

Estimar a relação entre gasto catastrófico em saúde (GCS) e multimorbidade em amostra nacional representativa da população brasileira com 50 anos de idade ou mais.

**MÉTODOS:**

Foram utilizados dados de 8.347 participantes do Estudo Longitudinal da Saúde dos Idosos Brasileiros (2015–2016). A variável dependente foi o GCS, definido pela razão entre as despesas com saúde do adulto de 50 anos ou mais e a renda domiciliar. A variável de interesse foi a multimorbidade (duas ou mais doenças crônicas), e a variável utilizada para estratificação foi o escore de riqueza. As principais análises foram baseadas na regressão logística multivariada.

**RESULTADOS:**

A prevalçncia de GCS foi de 17,9% e 7,5% para gastos correspondentes a 10% e 25% da renda domiciliar, respectivamente. A prevalçncia da multimorbidade foi de 63,2%. A multimorbidade apresentou associações positivas e independentes com GCS (OR = 1,95, IC95% 1,67–2,28 e OR = 1,40, IC95% 1,11–1,76 para gastos correspondentes a 10% e 25%, respectivamente). Os gastos associados à multimorbidade foram maiores entre aqueles com menor escore de riqueza.

**CONCLUSÕES:**

Os resultados chamam atenção para a necessidade de uma abordagem integrada da multimorbidade nos serviços de saúde, de forma a evitar os GCS, particularmente entre adultos mais velhos com piores condições socioeconômicas.

## INTRODUÇÃO

Nas últimas décadas, a preocupação relacionada aos gastos com saúde tem sido destaque globalmente, considerando que esses gastos tçm crescido mais rapidamente do que o Produto Interno Bruto (PIB) da maioria dos países, especialmente naqueles de baixa e média renda ^1^ . Entretanto, um maior gasto nacional não implica necessariamente melhores condições de saúde nem maior equidade no acesso aos serviços ^2^ . De maneira geral, os sistemas de saúde devem permitir que as pessoas utilizem os serviços sem incorrer em sacrifício financeiro, o que faz parte do objetivo de cobertura universal em saúde, proposto pela Organização das Nações Unidas, em seus Objetivos do Desenvolvimento Sustentável ^3^ . Embora muitos países se esforcem neste sentido, dados recentes apontam uma tendçncia de aumento dos gastos catastróficos com saúde ^4^ , aqueles que excedem uma porcentagem da renda, do consumo ou da capacidade de pagamento das famílias ^3^^–^^5^ . Existem diferentes pontos de corte, que variam de 10% a 40%, para definir esse tipo de gasto, a depender do denominador utilizado para o cálculo ^3^^–^^5^ .

Dados reportados pela Organização Mundial da Saúde (OMS) mostram que, em 2010, 808 milhões de pessoas (11,7% da população mundial) incorreram em gastos com saúde que excederam 10% do orçamento familiar e, para 179 milhões de pessoas (2,6% da população mundial), os gastos correspondentes excederam o limite de 25% do orçamento familiar ^3^ . No Brasil, entre 2007 e 2015, 25,6% da população incorreu em gastos com saúde superiores a 10% do total das despesas ou rendas domiciliares ^6^ .

Estudos apontam maior impacto dos gastos catastróficos com saúde em famílias de nível socioeconômico mais baixo ^7^^,^^8^ . Além disso, evidçncias mostram associação positiva entre doenças crônicas e maior desembolso direto com saúde ^9^^,^^10^ . Dentro do contexto de envelhecimento populacional, caracterizado pela predominância e pelo grande ônus das doenças crônicas não transmissíveis ^11^ , a preocupação com o impacto financeiro gerado por elas também tem sido objeto de estudo ^9^^,^^12^ .

O fenômeno da multimorbidade – a ocorrçncia simultânea de mais de uma doença no mesmo indivíduo ^13^ – também é cada vez mais comum, e com tendçncia de crescimento ^14^ . Idosos com multimorbidade representam uma complexa demanda, já que utilizam com maior frequçncia os serviços ^12^ , elevam os custos para os sistemas de saúde ^12^^,^^15^ e aumentam as chances de gastos catastróficos para as famílias ^9^ .

A relação entre condições socioeconômicas, doenças crônicas e gastos em saúde na população geral já foi avaliada por diferentes estudos ^7^^–^^10^ , porém a associação entre gasto catastrófico em saúde e multimorbidade em idosos ainda é pouco explorada, especialmente em países em desenvolvimento. No Brasil, essa associação é desconhecida, embora particularmente relevante, considerando o rápido envelhecimento populacional e as desigualdades sociais nas condições de saúde e no uso dos serviços dessa população ^16^ . Assim, os objetivos deste estudo são: (1) descrever a distribuição dos gastos catastróficos em saúde (GCS) em amostra nacional representativa da população brasileira de adultos mais velhos; (2) estimar a associação destes gastos com multimorbidade; e (3) avaliar se as condições socioeconômicas interferem nessa associação.

## MÉTODOS

Foram utilizados dados da linha de base do Estudo Longitudinal de Saúde dos Idosos Brasileiros (ELSI-Brasil), conduzido entre 2015 e 2016 em amostra representativa da população brasileira não institucionalizada, com idade igual ou superior a 50 anos. O ELSI-Brasil utilizou uma amostra probabilística selecionada por meio de processos de estratificação e conglomerados, em diferentes estágios. Nos domicílios selecionados, todos os residentes com 50 anos ou mais foram elegíveis para a entrevista e outros procedimentos. A amostra final da linha de base do ELSI-Brasil foi composta por 9.412 indivíduos, residentes em 70 municípios de diferentes regiões do país.

No presente estudo, foram considerados todos os indivíduos com idade igual ou superior a 50 anos que participaram do inquérito da linha de base da pesquisa e que possuíam todas as informações necessárias para a análise de dados proposta, totalizando 8.347 pessoas. Mais detalhes sobre o ELSI-Brasil podem ser vistos na *homepage* da pesquisa (elsi.cpqrr.fiocruz.br) e em publicação anterior ^17^ .

O ELSI-Brasil foi aprovado pelo Comitç de Ética em Pesquisa do Instituto René Rachou da Fundação Oswaldo Cruz (CAAE:34649814.3.0000.5091) e todos os participantes assinaram o Termo de Consentimento Livre e Esclarecido (TCLE) antes das entrevistas.

### Variável Dependente

A variável dependente do estudo foi o gasto catastrófico em saúde (sim/não), definido como a razão entre o desembolso direto, relativo a todas as despesas com saúde realizadas pelo adulto com 50 anos ou mais nos últimos 30 dias e a renda total do domicílio. Os indivíduos cujos gastos atingiram valores iguais ou maiores a 10% ou valores iguais ou maiores a 25% da renda domiciliar foram classificados como incorrendo em gastos catastróficos, conforme proposto previamente ^3^^,^^4^ .

Foram consideradas as seguintes despesas de saúde para compor o desembolso direto: consulta médica, consulta com dentista, internações hospitalares, fisioterapeuta, terapeuta ocupacional, fonoaudiólogo, psicólogo, cuidador/técnico de enfermagem, exames de laboratório/imagem e outros, nutricionista e medicamentos. No ELSI-Brasil, considerou-se o período recordatório de 90 dias para os gastos com saúde, com exceção das despesas com medicamentos, coletadas com base nos últimos 30 dias. Desta forma, para permitir a soma dos valores e calcular o desembolso direto nos últimos 30 dias, os gastos avaliados com período recordatório de 90 dias foram divididos por trçs. Todas as variáveis relacionadas ao gasto e à renda foram deflacionadas para o ano de 2015, utilizando-se o Índice Nacional de Preços ao Consumidor Amplo (IPCA) disponibilizado pelo Instituto Brasileiro de Geografia e Estatística (IBGE). O poder de compra de 1 real de 2016 equivalia, em média, a 95 centavos do ano de 2015.

A renda domiciliar foi estabelecida pela soma dos rendimentos de todos os moradores, considerando cinco fontes: salário ou trabalho autônomo; aposentadoria ou pensão por morte; Bolsa Família, Benefício de Prestação Continuada, pensão alimentícia ou doação em dinheiro; aluguéis ou arrendamentos; e outras. Foi assumido que os dados faltantes para um ou mais itens representariam ausçncia de renda no item em questão. Os entrevistados que não relataram o valor exato do rendimento foram solicitados a informar o intervalo mais próximo, sendo utilizado o ponto médio do intervalo como valor do rendimento. Para o último intervalo de classe, o valor foi imputado considerando o ganho mediano dos demais moradores com rendimento nessa faixa, que informaram a renda na pergunta aberta.

### Covariáveis

A multimorbidade, definida como a presença de duas ou mais condições crônicas de saúde ^13^ , foi a principal variável independente. As doenças crônicas foram selecionadas de uma lista com 17 doenças, sendo elas: hipertensão arterial, diabetes, colesterol alto, infarto do miocárdio, angina, insuficiçncia cardíaca, acidente vascular encefálico (AVE), asma, doença pulmonar obstrutiva crônica (DPOC), artrite ou reumatismo, osteoporose, problema na coluna, depressão, câncer, insuficiçncia renal crônica, doença de Parkinson e doença de Alzheimer. A presença dessas doenças foi avaliada por meio de autorrelato, utilizando-se a pergunta: “Algum médico já disse que o(a) senhor(a) tem...”.

As demais variáveis independentes incluíram: idade (50–59, 60–69, 70–79 e 80 anos ou mais); sexo (feminino e masculino); proporção de idosos no domicílio; escolaridade (não estudou, 1–3, 4–7 e 8 ou mais anos de estudo); estado civil (sem ou com relação marital); escore de riqueza (1º, 2º, 3º, 4º e 5º quintis); número médio de medicamentos utilizados; plano de saúde (sim e não); e limitação funcional (sim e não).

O escore de riqueza, categorizado em quintis, foi definido através da análise de componentes principais ^18^ , usando informações sobre propriedade familiar de bens duráveis e características da habitação: bens domésticos (internet, televisão, televisão a cabo, geladeira, máquina de lavar roupa, lava-louças, máquina de secar roupa, computador, telefone de mesa, telefone celular, micro-ondas, motocicleta, carro) e características domésticas (empregada doméstica, parede de alvenaria ou parede de madeira, água encanada, rua pavimentada, banheiro).

A limitação funcional foi avaliada segundo o relato de dificuldades para realizar uma ou mais atividades básicas de vida diária (atravessar de um cômodo a outro, vestir-se, tomar banho, comer, deitar-se e/ou levantar da cama e usar o banheiro).

### Análise dos Dados

Foi realizada análise descritiva das variáveis do estudo e da proporção das despesas de saúde em relação ao desembolso direto. Utilizou-se a regressão logística univariada, seguida de regressão logística múltipla, para avaliar a associação entre o gasto catastrófico e cada variável independente. Foram incluídas no modelo múltiplo todas as variáveis com p-valor menor do que 0,20 e permaneceram no modelo final aquelas significativamente associadas ao desfecho. Os resultados foram expressos por meio do *odds ratio* e respectivos intervalos de confiança (IC95%). As probabilidades ajustadas de gasto catastrófico entre indivíduos com multimorbidade de acordo com o escore de riqueza foram calculadas a partir da inclusão do termo de interação entre a multimorbidade e o escore de riqueza. A análise dos dados foi realizada com o *software Stata* 14.0 ( *StataCorp College Station* , Estados Unidos), considerando-se os parâmetros amostrais e os pesos dos indivíduos.

## RESULTADOS

A Tabela 1 apresenta a análise descritiva da amostra, composta majoritariamente por mulheres (54%), adultos na faixa etária entre 50 a 59 anos (46,9%), com relação marital (63,2%) e 8 anos ou mais de estudo (37,4%). A maioria da população estudada apresentava multimorbidade (63,2%), não tinha limitação funcional (84,4%) e não possuía plano de saúde privado (74,1%). A prevalçncia de GCS foi de 17,9% para o ponto de corte de 10% da renda domiciliar e de 7,5% para o ponto de corte de 25% da renda domiciliar.

**Tabela 1:** Características sociodemográficas e de saúde dos participantes da pesquisa (Estudo Longitudinal da Saúde dos Idosos Brasileiros), 2015–2016

Variáveis		%	IC95%
Sexo			
	Feminino	54,0	51,1–56,9
	Masculino	46,0	43,1–48,9
Faixa etária (anos)			
	50–59	46,9	42,6–51,3
	60–69	30,0	28,1–31,9
	70–79	15,8	14,0–17,8
	80+	7,3	6,0–8,8
Estado civil			
	Sem relação marital	36,8	33,9–39,8
	Com relação marital	63,2	60,2–66,1
Escolaridade			
	Não estudou	12,3	10,0–14,9
	1–3 anos	18,7	17,1–20,4
	4–7 anos	31,6	29,1–34,2
	8+ anos	37,4	34,6–40,4
Proporção de idosos no domicílio		70,7	69,2–72,2
Multimorbidade			
	Não	36,8	34,6–39,1
	Sim	63,2	60,9–65,4
Medicamentos (média)		2,2	2,1–2,3
Limitação funcional			
	Não	84,4	83,0–85,7
	Sim	15,6	14,3–17,0
Plano			
	Não	74,1	1,4–76,7
	Sim	25,9	23,3–28,6
Gasto catastrófico em saúde			
	Ponto de corte de 10%	17,9	16,4–19,5
	Ponto de corte de 25%	7,5	6,6–8,4

A partir da avaliação dos componentes do gasto em saúde, verificou-se que o gasto com medicamentos representou a maior proporção do desembolso direto (65,1%), seguido do gasto com dentista e do gasto com consultas médicas ( Tabela 2 ).

**Tabela 2:** Proporção média das despesas de saúde em relação ao desembolso direto de adultos mais velhos no Brasil (Estudo Longitudinal da Saúde dos Idosos Brasileiros), 2015–2016.

Despesas	Desembolso direto	
	%	IC95%
Medicamento	65,1	63,0–67,3
Dentista	12,2	10,7–13,7
Consulta médica	11,1	9,8–12,4
Exames	8,7	7,5–10,0
Fisioterapeuta	1,4	1,0–1,7
Cuidador	0,8	0,4–1,2
Internação	0,5	0,3–0,7
Psicólogo	0,5	0,3–0,6
Nutricionista	0,2	0,1–0,3
Terapeuta ocupacional	0,0	0,0–0,07
Fonoaudiólogo	0,0	0,0–0,1

Na análise univariada, todas as variáveis foram significativamente associadas ao gasto catastrófico nos dois pontos de corte, com exceção da escolaridade para ambos os pontos e da atividade física para o ponto de corte de 25% da renda domiciliar ( Tabela 3 ).

**Tabela 3:** Análises univariadas entre o gasto catastrófico e variáveis independentes (Estudo Longitudinal da Saúde dos Idosos Brasileiros), 2015–2016.

Variáveis		Gasto catastrófico	
		≥ 10% OR (IC 95%)	≥ 25% OR (IC 95%)
Sexo (ref. a : feminino)			
	Masculino	0,59 (0,51–0,70) b	0,53 (0,42–0,68) b
Idade (anos)		1,03 (1,02–1,04) b	1,02 (1,01–1,04) b
Relação marital (ref. a : não)			
	Sim	0,73 (0,64–0,82) b	0,63 (0,51–0,77) b
Anos de estudo		0,99 (0,98–1,01)	1,00 (0,98–1,02)
Escore de riqueza (ref. a : 1º quintil)			
	2º quintil	1,28 (1,02–1.60) c	1,36 (1,03–1,79) c
	3º quintil	1,16 (0,93–1,43)	1,09 (0,81–1,46)
	4º quintil	0,94 (0,72–1,23)	0,89 (0,63–1,24)
	5º quintil (mais ricos)	0,92 (0,69–1,23)	1,04 (0,72–1,49)
Proporção de idosos no domicílio		1,01 (1,01–1,01) b	1,01 (1,00–1,01) b
Atividade física (ref. a : não)			
	Sim	0,79 (0,67–0,93) d	0,84 (0,68–1,04)
Multimorbidade (ref. a : não)			
	Sim	2,45 (2,11–2,84) b	1,83 (1,47–2,28) b
Nº de medicamentos		1,34 (1,30–1,39) b	1,27 (1,22–1,32) b
Limitação funcional (ref. a : não)			
	Sim	2,45 (2,15–2.78) b	2,26 (1,78–2,87) b
Plano de saúde (ref. a : não)			
	Sim	1,36 (1,15–1,60) b	1,49 (1,17–1,88) b

a ref.: categoria de referçncia

b p < 0,001

c p < 0,05

d p < 0,01

A Tabela 4 apresenta os resultados dos fatores associados ao GCS. Após o ajuste do modelo, indivíduos do sexo feminino, mais velhos, com menor escolaridade, com limitação funcional e que possuíam plano de saúde privado apresentaram maiores chances de incorrer em GCS, para ambos os pontos de corte. Apenas a associação entre a escolaridade de 4 a 7 anos de estudo e o gasto igual ou maior a 25% da renda domiciliar e a associação entre o escore de riqueza para o mesmo ponto de corte não se mantiveram estatisticamente significativas após o ajuste do modelo. Observou-se ainda que a presença de multimorbidade aumentou em 95% a chance de GCS igual ou superior a 10%, além de aumentar em 40% a chance de GCS igual ou superior a 25% da renda domiciliar. Os resultados também apontaram que adultos mais velhos pertencentes ao maior escore de riqueza possuem 5% menos chances de incorrer em gasto catastrófico para o ponto de corte de 10% da renda domiciliar ( Tabela 4 ).

**Tabela 4:** Modelo múltiplo de regressão logística para os fatores associados ao gasto catastrófico em saúde (Estudo Longitudinal da Saúde dos Idosos Brasileiros), 2015–2016.

Variáveis		Gasto catastrófico	
		10% OR (IC 95%)	25% OR (IC 95%)
Sexo			
	Masculino	0,69 (0,58–0,81) a	0,59 (0,45–0,76) a
Idade		1,02 (1,01–1,03) a	1,02 (1,01–1,03) b
Escolaridade (8+ anos)			
	Não estudou	0,65 (0,50–0,85)^b^	0,64 (0,45–0,91) c
	1–3 anos	0,71 (0,57–0,89) b	0,75 (0,59–0,96) c
	4–7 anos	0,79 (0,67–0,93) b	0,82 (0,65–1,04)
Escore de riqueza		0,95 (0,92–0,98) b	0,96 (0,90–1,01)
Proporção de idosos no domicílio		1,01 (1,00–1,01) a	1,01 (1,00–1,01) b
Multimorbidade			
	Sim	1,95 (1,67–2,28) a	1,40 (1,11–1,76) b
Limitação funcional			
	Sim	2,06 (1,79–2,37)^a^	2,04 (1,62–2,56) a
Plano de saúde			
	Sim	1,28 (1,07–1,53) b	1,42 (1,11–1,82) b

a p < 0,001

b p < 0,01

c p < 0,05

A Figura 1 apresenta as probabilidades ajustadas de GCS de acordo com a presença de multimorbidade e escore de riqueza. Nos dois pontos de corte, verificaram-se diferenças significativas entre as probabilidades de gastos catastróficos de acordo com a presença de multimorbidade e o escore de riqueza. As probabilidades de GCS foram maiores entre indivíduos com multimorbidade e com menor escore de riqueza, para os dois pontos de corte.

**Figura:** Probabilidades ajustadas de gasto catastrófico em saúde entre adultos mais velhos com e sem multimorbidade de acordo com escore de riqueza (Estudo Longitudinal da Saúde dos Idosos Brasileiros), 2015–2016.

## DISCUSSÃO

O presente estudo verificou um impacto significativo das despesas com saúde na renda domiciliar de adultos mais velhos. Aproximadamente um quinto dos indivíduos analisados incorreram em GCS excedendo em 10% a renda domiciliar, e 8% dos indivíduos incorreram em GCS, quando utilizado o ponto de corte de 25%. O gasto com medicamentos foi a principal despesa com saúde. Os GCS foram significativamente associados à multimorbidade, a qual potencializou também os efeitos das desigualdades socioeconômicas, independentemente do ponto de corte utilizado.

A prevalçncia de GCS estimada para os dois pontos de corte é superior à encontrada na população total de países ricos e de países da América Latina e Caribe ^4^ , onde a estimativa de GCS alcançou 13,4% em 2000, saltando para 14,8% em 2010, considerando o total de despesas com saúde da família para o ponto de corte de 10% do consumo familiar ^4^ . Entre países ricos, estudo realizado com australianos de 55 anos ou mais verificou que 11,8% e 5,1% incorreram em GCS para os pontos de corte de 10% e 20%, respectivamente ^9^ . Ante a estimativa de 25,6% da OMS, para o período entre 2007 e 2015, que considerou a população brasileira como um todo ^6^ , a prevalçncia de GCS entre adultos mais velhos no país para o ponto de corte de 10% pode ser considerada elevada.

A maior prevalçncia de GCS entre idosos, em relação ao total da população, tem sido descrita na literatura ^19^ , podendo ser explicada pelas diferenças nos tipos e frequçncia de gastos entre famílias jovens, quando comparadas às famílias de idosos. Em geral, a maior parte das despesas com saúde se refere aos gastos com medicamentos e consultas médicas ^20^ . Entre idosos, além destes gastos serem mais elevados, a sua proporção em relação à renda também é maior ^15^ .

Corroborando estudo prévio ^9^ , verificou-se que indivíduos com multimorbidade apresentaram mais chances de ter gastos catastróficos, independente do ponto de corte. McRae et al. ^9^ não só observaram associação positiva entre multimorbidade e GCS entre adultos mais velhos, como verificaram um gradiente nesta relação: cada doença crônica adicional aumentou em 46% a chance de gastos com saúde excederem 20% da renda ^9^ . Entre os motivos para esta associação, pode-se mencionar a maior utilização dos serviços por indivíduos com multimorbidade ^12^ , a qual está relacionada à complexidade do cuidado, como por exemplo, a necessidade de utilização de vários medicamentos ^21^ . Indivíduos com multimorbidade podem gastar em média 10 vezes mais com saúde do que aqueles sem multimorbidade ^10^ .

Os resultados do presente estudo também demostram que há desigualdades socioeconômicas relacionadas ao GCS, relação consistentemente descrita na literatura ^8^^,^^22^ . No Brasil, a ocorrçncia de GCS para a população total foi 5,2 vezes maior entre os mais pobres, segundo o Índice Econômico Nacional, e 4,2 vezes maior entre os indivíduos com menor escolaridade, segundo anos de estudos completos ^8^ . O presente estudo avança ao mostrar que as desigualdades associadas aos GCS entre adultos mais velhos apresentam padrões distintos, quando avaliadas em função da multimorbidade, que potencializa o efeito dessas desigualdades. Independentemente do ponto de corte utilizado, a probabilidade de GCS foi maior entre indivíduos com multimorbidade e pertencentes aos menores escores de riqueza, quando comparados aos indivíduos sem multimorbidade, sendo esta diferença significativamente atenuada com o aumento da condição socioeconômica.

Ambos resultados reforçam a importância do investimento para efetivação de uma cobertura universal em saúde que garanta acesso a serviços e cuidados de qualidade, sem comprometer de forma catastrófica o orçamento das famílias ^3^ . No Brasil, a manutenção do Sistema Único de Saúde (SUS) é essencial para que este objetivo seja alcançado, pois ao longo dos seus 30 anos de existçncia, o sistema se mostrou efetivo na redução de desigualdades no acesso e nas condições de saúde da população ^23^ , especialmente entre indivíduos com doenças crônicas, considerando que mais de 70% dos idosos brasileiros dependem exclusivamente do SUS ^24^ . Além disso, dados da mais recente pesquisa nacional sobre acesso a medicamentos no Brasil mostraram que 67,7% dos adultos e idosos brasileiros com doenças crônicas que tiveram acesso total ao tratamento, obtiveram algum medicamento gratuitamente, sendo que 47,5% obtiveram todos os medicamentos dessa forma ^25^ .

Ainda que o acesso a medicamentos gratuitos no SUS seja maior entre indivíduos com baixa renda, pessoas de média renda também costumam acessar a assistçncia farmacçutica pública ^26^ . Isso pode ter relação com o tipo de plano e seguro de saúde privados contratados por esta parcela da população. Estes planos comumente apresentam cobertura limitada para medicamentos, atendimento domiciliar, fisioterapia e outros serviços, fazendo com que indivíduos que possuem este tipo de plano privado de saúde recorram ao sistema público para garantir o acesso à estes serviços ^27^ . Estudo recente demostrou a utilização exclusiva do SUS para obter medicamentos por 29,3% dos indivíduos com doenças crônicas que possuíam plano de saúde e entre 34,4% daqueles com estas doenças e que pertenciam a maior classificação econômica ^27^ . Esta pode ser uma das explicações para o fato de o plano privado de saúde não constituir um fator de proteção contra gastos catastróficos em saúde ^7^ , podendo inclusive aumentar a ocorrçncia deste desfecho, conforme apontam os resultados do presente estudo. Assim como no Brasil, outros estudos recentes realizados em países como o México ^28^ e a China ^29^ demonstraram que programas públicos de saúde diminuem os riscos de gastos catastróficos.

Dentre os pontos fortes deste estudo, destaca-se a utilização de uma medida de gasto em saúde composta por um número abrangente de despesas, normalmente indisponível em estudos populacionais, possibilitando uma análise mais específica dos gastos realizados por adultos mais velhos no Brasil. No entanto, o autorrelato de doenças crônicas pode interferir na estimativa da multimorbidade, embora esta medida seja considerada válida em estudos epidemiológicos, sem a ocorrçncia de grande viés socioeconômico ^30^ . O fato deste estudo ter utilizado o desembolso direto no numerador, bem como a renda domiciliar no denominador, em detrimento do gasto total em saúde e da capacidade de pagamento da família, pode ter reduzido o percentual de GCS. Embora tais medidas sejam amplamente utilizadas nas pesquisas, não há consenso na literatura sobre a melhor forma de cálculo. O delineamento transversal constitui outra limitação deste estudo, na medida em que impede a análise das relações temporais entre as variáveis. O período recordatório das medidas utilizadas para o cálculo do GCS, restrito a 30 e 90 dias, também pode ter interferido na magnitude do desfecho.

Os resultados deste estudo ajudam a entender o gasto catastrófico em saúde entre adultos mais velhos no Brasil, que pode ser potencializado pela presença de multimorbidade e entre indivíduos com baixa condição socioeconômica. Considerando o envelhecimento populacional, acompanhado de um aumento na prevalçncia de multimorbidade ^14^ , os sistemas de saúde precisam se adaptar a esta demanda, garantindo o acesso e a qualidade de seus serviços, especialmente aos grupos populacionais menos favorecidos. Pesquisas futuras são necessárias para monitorar os achados aqui apresentados e investigar a influçncia de combinações específicas de doenças sobre a ocorrçncia de GCS, com o objetivo de contribuir para a avaliação do impacto de políticas de cobertura universal em saúde na redução das desigualdades.
